# Real-world application, challenges and implication of artificial intelligence in healthcare: an essay

**DOI:** 10.11604/pamj.2022.43.3.33384

**Published:** 2022-09-02

**Authors:** Shiv Kumar Mudgal, Rajat Agarwal, Jitender Chaturvedi, Rakhi Gaur, Nishit Ranjan

**Affiliations:** 1 College of Nursing, All India Institute of Medical Sciences, Deoghar, Jharkhand, India,; 2 Department of Cardiothoracic Surgery, All India Institute of Medical Sciences, Deoghar, Jharkhand, India,; 3 Department of Neurosurgery, All India Institute of Medical Sciences, Rishikesh, Uttarakhand, India

**Keywords:** Artificial intelligence, application, challenges, real-world, healthcare

## Abstract

This essay examines the state of Artificial Intelligence (AI) based technology applications in healthcare and the impact they have on the industry. This study comprised a detailed review of the literature and analyzed real-world examples of AI applications in healthcare. The findings show that major hospitals use AI-based technology to enhance knowledge and skills of their healthcare professionals for patient diagnosis and treatment. AI systems have also been shown to improve the efficiency and management of hospitals´ nursing and managerial functions. Healthcare providers are positively accepting AI in multiple arenas. However, its applications offer both the utopian (new opportunities) as well as the dystopian (challenges). Unlike pessimists, AI should not be seen a potential source of “Digital Dictatorship” in future of 22^nd^ century. To provide a balanced view on the potential and challenges of AI in healthcare, we discuss these details. It is evident that AI and related technologies are rapidly evolving and will allow care providers to create new value for patients and improve their operational efficiency. Effective AI applications will require planning and strategies that transform both the care service and the operations in order to reap the benefits.

## Essay

### Introduction

Artificial Intelligence (AI), a technology prevalent for almost 60-year has made it possible to create applications that have a profound effect on our life today. It seeks to reproduce and modify human intelligence leading to development of intelligent machines [1]. Some researchers believe that AI can think and act rationally. Others disagree that AI is capable of acting and thinking like humans. Irrespective of what anyone believes, it appears for sure that in the year 2100, the health industry is expected to survive on AI-Human cooperation, not competition. Artificial intelligence, a broad-based tool, allow humans to rethink the way they integrate information, analyze data and use the insights to improve their decision-making. It is already transforming all walks of life [2].

AI is not something futuristic, but a technology that is already in use and integrated into many sectors. Examples include public healthcare and education, transport, telecommunications, data security management, finance, research, policymaking and the legal and judiciary system. AI technologies are now being increasingly applied to healthcare [3]. A combination of unstoppable forces drives healthcare demand. These include changing patient expectations, increasing population age, lifestyle shifts, and the never-ending circle of innovation. The Healthcare system must undergo significant structural and transformational changes to ensure its sustainability. AI has potential to transform healthcare and address some of these challenges [4,5].

AI has been welcomed by healthcare systems around the world, which struggle to fulfil the “quadruple objective” of improving the health and well-being of their patients, healthcare access, cost-effectiveness [6] and improving the lives of healthcare workers [7]. It is essential for healthcare providers to be well versed in the potential applications of AI technologies in different aspects of healthcare which may embark digital revolution in this sector [8]. This article will discuss numerous applications and issues of AI technology in the healthcare industry in the present times. The article also serves necessary recommendations which will help healthcare managers with strategic planning and execution of AI in healthcare.

### Operational terms

#### What Is AI?

UNESCO defines AI systems as “technological systems that can process information in a manner that resembles intelligent behavior” [9]. A simplified definition of AI for healthcare is the ability to use computer programs to perform tasks or reasoning in multiple areas of healthcare, including diagnosis and treatment. This is similar to the intelligence that we associate with intelligence in humans [10]. AI in healthcare also refers to the use of machine-learning algorithms or software to replicate human cognition in the analysis and presentation of complex medical and healthcare data [11].

### Types of AI

**Figure 1:**
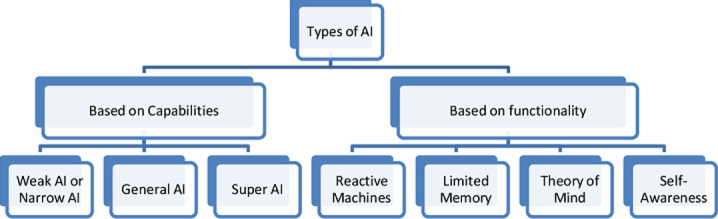
types of artificial intelligence

The main categories of AI are based on the capabilities and functions of AI. The types of AI are explained in the diagram below (Figure 1).

### Based on capabilities of AI-based systems

i) Weak AI or Narrow AI: an AI that can perform a specific or a limited set of tasks without any thinking abilities. The Weak AI category covers almost all AI-based systems that have been developed to date such as Siri, Alexa Self-driving car, Alpha-Go, Sophia the humanoid and speech recognition agent [12,13]. ii) General AI or Strong AI - It can perform any intellectual task as efficiently as a human. Although there are no examples of Strong AI to date, we believe that it will soon become possible to build machines as smart and intelligent as humans [12,13]. iii) Super AI: this level of intelligence of systems is at which machines can surpass human intelligence and have cognitive properties. It is currently a fictional scenario [12,13].

### Based on the functionality of AI-based systems

i) Reactive machine AI: this category of AI includes machines that work solely from current data and take only current circumstances into consideration. It cannot draw inferences to predict their future actions. They can do a narrower variety of pre-defined functions. Examples include Google's Alpha Go and IBM's Deep Blue systems [12,13]. ii) Limited Memory AI: this has limited memory. It can make better decisions by looking at past data, can store past experiences in a short-term or temporary manner and then use that information to predict future actions. Example includes self-driving vehicles [12,13]. iii) The Theory of Mind AI: this AI should understand people, emotions, and beliefs of human beings, and can interact with them socially as humans. This type of AI machine is not yet developed [14,15]. iv) Self-aware AI: self-awareness AI is the future. This technology will build super-intelligent machines that will possess their own consciousness, feelings, and self-awareness. These machines will be more intelligent than the human brain. It´s only a fictional scenario at present [14,15].

### The state of AI technology

Artificial intelligence does not refer to one technology. Many AI technologies can be applied immediately to healthcare. Some great AI technologies in healthcare are mentioned below:

***Machine learning (ML)*:** machine learning is the dominant approach to AI. It uses a predictive model for making predictions from predefined data. Machine learning is being used in many AI technologies, such as natural language processing (NLP) and voice technology [8]. Supervised learning, reinforcement learning (RL), deep learning and multi-instances learning are some of the most popular ML algorithms [14].

***Supervised learning*:** this approach uses a set of data and known, defined outcomes as an outcome. Then, patterns are identified that correspond with the input to make predictions. The algorithm must know what conclusions it should draw from the given data set. Healthcare has witnessed a lot of supervised learning. This allows for data-driven clinical decisions to be made, e.g., use of imaging to diagnose tumours and determine their severity, and predictive analytics within continuous outputs e.g., use of EHR to predict the recurrences, prognosis and mortality of a particular disease [14,15].

***Unsupervised learning*:** this approach can find the data structure and forecast based only on that input. It is better suited for uncertain outcomes or when data labelling is expensive. Unsupervised learning can be used in healthcare to predict individual disease risks and design personalized treatments that are based on genetic biomarkers and genomic variations [8,15].

***Semi-supervised learning*:** unsupervised learning is able to learn by itself, without any human interventions for the outcome. Unsupervised learning, even without human instruction, can be more susceptible to errors as it may use minor features of the data for predictions. In practice, semi-supervised learning is often used. It uses a combination of large untagged and small tagged data for training [15].

***Reinforcement learning*:** it is an autonomous algorithm that allows the user to act and interact according to the environment. It is one of the best learning models and very effective for tasks with clearly defined protocols. It relies on its own experience using feedback from mistakes and rewards to lead training. It does not require data or labelling. It is useful in healthcare such as optimizing treatment plans and robotic-assisted surgical procedures [15,16].

***Deep learning*:** deep learning uses a backpropagation algorithm that operates on multiple levels of abstraction to uncover the complex structure of large datasets. This algorithm is designed for the solution of difficult practical world issues. Some examples are: computer vision, Go game, speech recognition, NLP, genomics and drug discovery [8, 16].

***Natural language processing*:** this employs a computational approach to automatically interpret and represent human language, mainly in text form. These include machine translation, speech recognition, speech classification, question answering and sentiment analysis. Natural language processing tools can extract vital information about patients from large textual data like doctor´s prescriptions, daily patient notes, discharge summaries and various radiological / laboratory reports. This can help healthcare providers in speedy management of patients thus optimizing the health care delivery [8, 16].

***Real-world applications*:** the meaningful and practical application of AI, provides healthcare providers with opportunities and confidence to boost their skills to new challenges in healthcare.

### Real-World AI applications in healthcare

Some of the important practical applications in healthcare are mentioned in the following sections:

***Precision medicine*:** one of the most important applications of AI in healthcare is precision medicine. Precision medicine aims to optimize the path for diagnosis, therapeutic intervention and prognosis. It uses large multidimensional biological data sets that capture individual variability in genes and other contributing factors like age, gender, and race, as well as medical treatment options such as immune profile, metabolism and vulnerability to the environment. This allows clinicians to tailor early interventions, whether preventative or treatment-oriented, to each patient. There are many precision medicine initiatives [17]. These can be divided into three categories: digital health apps and complex algorithms, as well as genomic-based tests [17]. A deep learning algorithm was developed in collaboration with Scripps Research Institute (CA, USA) and Intel. With a precision of 85%, it could identify 23 patients at high risk for cardiovascular disease. This cognitive assistant is equipped with clinical knowledge and reasoning [18].

***Improved disease treatment*:** AI technologies are increasingly adding to the support of healthcare workers in various aspects of patient´s management. For instance, Onduo offers virtual coaching on mobile apps to control diabetes. It employs AI technology to detect food, and monitor glucose levels as well as physical activities, in order to make recommendations. DayTwo provides another solution for diabetes management. It provides an individualized meal suggestion, based on the user's gut microflora for adequate blood sugar levels. The recommended diet is chosen from its large index of over 100,000 foods [19]. ResApp Health, another example of AI used in chronic disease management, analyzes subjects' breathing by using their phone microphone. The AI algorithm then evaluates various respiratory conditions like chronic obstructive lung disease, pneumonia accurately [20].

***Improved diagnostic error reduction and decision support:*** AI will be used to aid in diagnosing patients with certain diseases and reduce human errors. AI was used by the Mayo Clinic to screen for cervical cancer in order to detect pre-cancerous changes. To identify precancerous signs, the AI-based algorithm uses over 60,000 images of cervical cancer from the National Cancer Institute. The accuracy rate of the algorithm was 91% as compared to 69% by skilled human expert [21]. The focus of IBM's Watson for Oncology has been a focus of media, especially in oncology management. Watson uses combination machine learning and NLP capabilities [22]. Freenome, which uses molecular biology and machine learning to detect early-stage cancers, is another example. The model can be trained to identify which biomarker patterns indicate the stage, type and best treatment options for particular cancer. AI can be used to detect disease-associated patterns by decoding hidden patterns. Google health uses AI for breast cancer screening. It demonstrated that its AI system can outperform human experts in breast-cancer prediction [23]. A deep learning-based AI developed by Massachusetts Institute of Technology (MIT), can forecast the possibility of development of breast cancer up to five years ahead [23].

London´s Moorefield´s Eye Hospital, has declared an AI solution for identifying ocular disease. The AI-based algorithm used data from greater than 15,000 British patients to detect ocular diseases by optical coherence. The decision of referral made by the AI-based algorithm was 94% accurate [24]. Google's research team developed a deep learning algorithm that can interpret retinal images to identify signs of diabetic retinopathy. This could potentially help doctors screen more patients in areas with fewer resources [18]. There are between 6000 and 8000 rare diseases that affect approximately 400 million people around the globe. A rare disease diagnosis can take up to five years and is often time-consuming having a great impact on the finances of the patient and the system. 3Billion created an algorithm in 2019 to diagnose rare DNA-based conditions which can test for up to 7000 diseases simultaneously in suspected cases [25].

***Patient data analytics*:** AI allows hospitals for clinical data analysis which can provide in-depth of patient´s health. It can also be used to predict prognosis, help in clinical audits, track patient prescription and refills, predict the advantages of specific drugs and identify patients´ at risk for substance abuse [26]. For example, the Paris public university hospital uses the Intel analytics platform for predicting the number of patients visiting the emergency department [27-29]. The potential volume of data is huge. According to estimates, personal lifestyle-based data amount to approximately 1100 terabytes in a lifetime. Genetics and medical data account for 6.4 terabytes. Omics technology, GWAS and EWAS, smartphone-based digital phenotyping, sensors and EHRs, and wearable devices can accurately monitor the lifestyle of a person along with climate and topographical data. This made it possible to implement strategies for the prevention and management of metabolic lifestyle disorders. This is why structured data collection and analysis are necessary for large, multidimensional studies which can be employed by integration ML/AI in healthcare system [29,30].

***Medical robotics*:** medical robots have many uses. They can be used to assist in surgery, in rehabilitation for stroke patients (rehabilitation robotics), care for elderly persons (assist-living robotic companion) social interaction (humanoid robot) and so on. AI-assisted surgeon robots have found their way into operation theatres. They can perform surgeries without fatigue and very useful at places where human hands cannot operate due to space constraints [27,31]. The Da Vinci is a surgical robot that allows professionals to perform complex procedures with greater flexibility and control than traditional approaches. The Da Vinci is a surgical robot that can assist surgeons by translating their hand movements at the console and creating clear, magnified, 3D high-resolution images of the surgical site [32].

***Real-time prioritization and triage*:** triage machine learning has been shown to be an efficient tool. John Hopkins University researchers found that ML-based e-triage improves patient risk assessment and categorization. Enlitic is patient triaging software that prioritizes cases according to their clinical data and directs them to suitable medical personnel [27]. Babylon health provides applicable health and triage information depending upon symptoms of the patient [33].

***Personalized care or virtual assistance*:** the treatment plans based on patient data reduce cost and increase the effectiveness of care. Human-Machine Interfaces (HMIs) analyze and recognizes facial motions and helps person with disabilities to drive robotic vehicles and wheelchairs [34]. RUDO, an “ambient intelligent system”, can be used to help blind people live with sighted people and work in trained fields like computer science. Blind people can access the various functions of the virtual assistant through a single interface [35]. An AI-based smart assistant can advise pregnant mothers about various important antenatal matters. AI applications can help the elderly with routine medications and can predict and prevent falls. This can be of major help in patients with gait disorders like Parkinson´s disease [28]. Chatbots allow patients to self-diagnose or help physicians in making a diagnosis. They can help patients share their health information in a proactive way. This allows medical professionals to improve quality care with cost-effectiveness. It also helps to increase patient satisfaction [27]. GYANT is a chatbot for healthcare that helps patients understand their symptoms. Doctors then receive the data and can diagnose and prescribe medicines in real time. Woebot is another chatbot that focuses on mental health. It calls itself “the next generation of mental health” and it certainly seems that way. The chatbot uses Cognitive Behavioral Therapy or CBT, to listen and offer advice to anyone who seeks it out [36]. AI apps that monitor and assist patients´ compliance to prescribed medication and treatment have been proven to be effective. Sentrian uses AI to analyze the data collected from patients' sensors at home. The goal is to identify signs and conditions that could lead to deterioration early so that intervention can be taken to prevent hospital admissions [37].

***Virtual assistants for nursing*:** AI virtual assistants are great in nursing because they can keep healthcare providers and patients connected all the time and thus decreases pressure on the already overburdened medical staff. Alexa robots are virtual nursing assistants employed by Cedars-Sinai Hospital in Los Angeles, California help nursing staff in their daily chores. [22]. Sensely, a virtual nurse, use Natural Language Processing, Machine Learning and wireless integration to medical devices, such as blood pressure monitors, to provide assistance to patients. Sensely can help you with self-care and clinical advice. It also helps you to schedule an appointment [38].

***Administrative workflow assistance*:** one of the AI applications in healthcare is the automation of administrative workflow. AI systems are able to perform operations like the transcription of medical records, medical billing services, bed allotment, and insurance claim verifications apart from numerous other hospital administrative activities faster and much more accurately than humans.[22,38] faster and better than individuals. The AI robot Paul accompanies the medical personnel in their daily patient rounds, help in the analysis of patient medical records and can provide any information regarding patient including daily investigations in a fraction of a second. Maria, the robot's guide, provides directions to patients in the hospital lobby to their doctor's offices or specific medical departments within the hospital and schedules appointments by touching the robot with their medical ID card [39]. The official statement made by Johns Hopkins University Hospital regarding AI technology stated: “Emergency room patients are assigned beds 30% faster, transfer delays from operating rooms are reduced by 70%, ambulances can pick up patients from other hospitals 63 minutes earlier, and the ability to take patients with complex medical conditions from regional and national hospitals has improved to 60%” [40]. Microsoft´s AI digital Assistant Cortana employed advanced analytics and predictive technology to identify potential patients at-risk in ICU treatment and able to monitor “100 beds in 6 ICUs”. [40].

***Improved operational efficiency and cost effectiveness*:** AI-based medical systems can perform numerous tasks involved in healthcare services in a simplified and cost-effective manner. Some of the tasks can be even done without human support. An AI-integrated pill-cam can substitute conventional upper endoscopy. Escalante *et al*. developed an AI-based method for diagnosis of acute leukemia by examining bone marrow structure characteristics non-invasively [41].

***Improving biomedical research*:** AI acts as an “eDoctor” to diagnose, manage, and prognosis diseases. AI can be a great tool for the indexing of medical literature. It can be used to formulate a research question, search available literature within seconds and test scientific hypotheses. This can save a lot of time and allow the researchers to perform good studies with relevant conclusions in shortest possible time [28,42].

***Drug discovery*:** deep learning has many promising applications in drug discovery. These include advanced image analysis, prediction of molecular structure, function and automated generation of unique chemical entities, de novo drug design, prediction of drug activity, prediction of drug-receptor interactions and prediction regarding drug reaction [43]. NuMedii, a Biopharma firm, developed an AIDD technology (Artificial Intelligence for Drug Discovery), that can identify rapid connections between drugs, diseases, and systems, if any [27]. Researchers created Eve, an AI “robot scientist” that is meant to speed up the process of drug discovery in a more economical way [44].

### Potential challenges of the application of AI to the healthcare industry

Some of the most significant challenges in the widespread use of AI include:

***Data privacy and cyber security*:** privacy issues can arise when confidential patient data is collected and shared by AI-based systems/technologies on large datasets. Thus, it is important that AI technology must follow, medical ethics, and laws and should be governed by some laws [22]. The highly sensitive confidential data of patients can be accessed and manipulated by miscreants who may be detrimental to the patient´s social life. Also, there may be high chances of misdiagnosis because of wrong faked data by AI systems. One study showed that benign moles could be misdiagnosed as malignant simply by adding antagonistic noises or just rotation [45].

***Reliability and safety*:** any error made by AI system, if not rectified early can lead to wrong results of the assigned tasks which may have serious consequences. For example, an AI app used for predicting the likelihood of patients developing complications after pneumonia wrongly advised doctors to send asthmatic patients home [46].

***Accountability of technology use*:** if AI-based technology used by medical staff leads to the death of the patient, “who would be responsible for the outcome?” This will create multiple unanswered questions on many technical, managerial and ethical issues [22].

***Potential loss of support system and autonomy*:** AI health apps may empower individuals to manage their own symptoms and take care of their own needs as and when required. This can have a potential impact on the employment of healthcare workers. This can also lead to less dependency on family members and can lead to isolation and behavioural issues [47]. AI agents could affect individual autonomy negatively by narrowing the treatment options and thus restricting patients to make informed consent about the procedure [45].

***Challenges in generalization to new populations*:** AI systems are still far from being able to provide reliable generalizability or clinical application for most types of medical data [45].

***Technological challenges*:** AI models are usually developed by non-medical professionals and thus end users (healthcare providers and patients) have no control in the derivation of the results. This lack of transparency is one of the major challenges in front of government policymakers. Another challenge is AI technology's limitations as they are designed by humans and any minute error in designing AI system can lead to wrong results. In addition, AI systems are not able to handle unstructured information such as medical imaging, which makes up a significant chunk of medical data in healthcare. Lastly, there is no standardization of data which is to be fed into databases and this can lead to different results in different locations [47].

***Organizational and managerial challenges*:** there are various challenges in developing AI like exchange and possession of data along with the potential danger of losing skilled healthcare providers and ground-level workers [41].

***Malicious use*:** although AI can be used to benefit humanity, it is also susceptible to being used maliciously. AI can be used to covertly monitor and analyze motor behaviours that can reveal the identity and secret information of the involved person [43].

**Conclusion:** in today´s digital age, innovation is essential. AI and related technologies can be very useful adjuncts to healthcare leaders in various aspects of healthcare management. They should not be viewed as a substitute for medical personnel but as a growing necessity that industries must embrace in order to have a competitive advantage. Artificial Intelligence and Human Stupidity run side by side to improve life of none other than stupid humans. AI over shine its master in two important aspects: connectivity and updatability. Because of its transformative nature in healthcare, the healthcare industry is particularly subjected to the potential of AI applications. AI applications have the potential to change not only the treatment and diagnosis processes but also the lifestyles of patients. In this study, we examined the impact AI technology on healthcare, as well as the types of new challenges and opportunities it has provided. We also recommend the establishment of a legal and ethical structure for AI, and drawing a social consensus between all stakeholders.
